# Psychiatric events induced by roflumilast: a real-world pharmacovigilance study of the FDA Adverse Event Reporting System database

**DOI:** 10.3389/fpsyt.2026.1836593

**Published:** 2026-06-11

**Authors:** Lifang Wu, Yan Chen, Yaping Huang, Shaojun Jiang, Chengjie Ke

**Affiliations:** 1 Department of Pharmacy, Second Affiliated Hospital of Xiamen Medical College, Xiamen, China; 2 Department of Pharmacy, Pingtan Comprehensive Experimental Area Hospital, Pingtan Comprehensive Experimental Area, Fuzhou, China; 3 Department of Pharmacy, Fujian Provincial Hospital, Fuzhou University Affiliated Provincial Hospital, Fuzhou, China; 4 Department of Pharmacy, The First Affiliated Hospital, Fujian Medical University, Fuzhou, China; 5 Department of Pharmacy, National Regional Medical Center, Binhai Campus of the First Affiliated Hospital, Fujian Medical University, Fuzhou, China

**Keywords:** FAERS, pharmacovigilance study, psychiatric events, roflumilast, safety

## Abstract

**Background:**

Roflumilast has approved treatment in patients with chronic obstructive pulmonary disease. The post-marketing data concerning roflumilast remain limited, and the outcomes of relevant studies are yet to yield conclusive evidence supporting the long-term psychiatric safety of roflumilast.

**Methods:**

This investigation comprehensively assessed psychiatric adverse events (AEs) attributed to roflumilast by employing data mining techniques, utilizing the FDA Adverse Event Reporting System (FAERS). The dataset encompasses the period from the first quarter of 2014 to the first quarter of 2025. A disproportionality analysis was conducted to quantify the correlation between roflumilast and psychiatric AEs. The metrics employed for the evaluation of disproportionality comprise the reporting odds ratio (ROR), the proportional reporting ratio, the information component, and the empirical Bayesian geometric mean.

**Results:**

A total of 414 reports were identified as related to psychiatric AEs of roflumilast, with the identification of 31 preferred terms. Five of psychiatric AEs exhibited significant positive signals, including insomnia (ROR 3.55), suicidal ideation (ROR 4.32), sleep disorder due to a general medical condition (ROR 13.47), nervousness (ROR 3.30), and eating disorder (ROR 5.16). A relatively increases risk of life-threatening and hospitalization in psychiatric AEs.

**Conclusion:**

Our findings would provide valued evidence for healthcare professionals to recognize psychiatric AEs associated with roflumilast and guide their clinical practice.

## Highlights

- 414 reports of roflumilast-related psychiatric AEs and 31 preferred terms were identified.
- There were 5 significant positive signals found in this study, such as insomnia, suicidal ideation, sleep disorder, nervousness, and eating disorder.
- Our research would provide valuable evidence for healthcare professionals to mitigate the risk of roflumilast-related psychiatric AEs, offering practical insights that can help clinical decision-making and patient management during roflumilast application.

## 1 Introduction

Chronic obstructive pulmonary disease (COPD) is a common respiratory disease characterized by emphysema, mucus hypersecretion, and persistent lung inflammation. The inflammatory environment of COPD is typified by the infiltration of the airways and lung tissue by inflammatory cells, including neutrophils, macrophages and lymphocytes (1). Globally, COPD ranks as the fourth leading cause of mortality, responsible for 3.5 million deaths in 2021, constituting nearly 5% of all deaths worldwide (2). The main objective in COPD treatment strategies is to alleviate symptoms, prevent complications and impede disease progression. Pharmacologic interventions predominantly include anticholinergics, glucocorticoids, β-adrenergic receptor agonists, and phosphodiesterase-4 (PDE4) inhibitors, which function to reduce inflammation and promote bronchodilation (3). Inflammation plays a crucial role in the development, progression, and aggravation of COPD-related pulmonary damage (4). PDE4 inhibition has emerged as a promising therapeutic strategy for COPD treatment, given that its wide range of anti-inflammatory effects *in vitro* and *in vivo (*5–7).

Rofumilast, a new PDE4 inhibitor, significantly reduces airway inflammation in patients with COPD, as reflected by reductions by sputum neutrophil and eosinophil counts (8). This reduces the rate of exacerbation and decline in lung function in COPD (9–12). In a randomized clinical trial, prebronchodilator forced expiratory volume in one second (FEV_1_) increased by 48 ml with roflumilast compared with placebo (*P* < 0.0001). The rate of moderate or severe exacerbations per patient per year was 17% lower with roflumilast than placebo (*P* < 0.0003) (10, 11). In another randomized trial, the roflumilast group exhibited a 13.2% reduction in the rate of moderate-to-severe COPD exacerbations compared to the placebo group (11).

The most common adverse events (AEs) associated with roflumilast are COPD exacerbations, diarrhea, pneumonia, nausea, headache, weight decrease, back pain, influenza, insomnia, dizziness, and diminished appetite (10, 11, 13). Serious AEs most frequently reported are COPD exacerbations and pneumonia, other serious AEs encompass diarrhea, atrial fibrillation, lung cancer, prostate cancer, acute pancreatitis, and acute renal failure (11, 13). Notably, roflumilast has been associated with an increased risk of psychiatric AEs, including suicidality, insomnia, anxiety, depression, suicidal thoughts or other mood changes (13, 14). Insomnia, anxiety, and depression are the most common of these, with approximately 2% of patients receiving roflumilast experiencing insomnia (9, 10, 12, 15–17). Previous study has reported that suicide-related AEs in five individuals undergoing roflumilast treatment (18). Another study also reported psychiatric AEs associated with roflumilast, such as insomnia and suicidality (19). The risk of psychiatric AEs associated with roflumilast treatment has increased, particularly with regard to the likelihood of experiencing sleep disturbances, anxiety, and depressed mood (20).

Although several studies have reported roflumilast-related psychiatric AEs, these finding are primarily derived from controlled clinical trials with small sample sizes. To date, post-marketing data concerning roflumilast remains limited, and the existing evidence has yet to conclusively evidence establish its long-term psychiatric safety. The linkage between roflumilast and psychiatric AEs remains uncertain, it is imperative to evaluate its post-marketing safety profile in the real-world settings. The Food and Drug Administration Adverse Event Reporting System (FAERS) is a valuable post-marketing surveillance database that can be used to identify drug safety concerns early on and assess the potential drug-AE associations (21, 22). In this study, a comprehensive pharmacovigilance analysis was performed using the FAERS database to explore the long-term psychiatric safety profile of roflumilast. This study aimed to investigated the relationship between roflumilast and psychiatric AEs by assessing signals, examining severe outcomes, evaluating time-to-onset, and conducting stratified analysis. Our findings are expected to bridge existing knowledge gaps and enhance post-marketing monitoring efforts concerning the psychiatric safety of roflumilast.

## 2 Methods

### 2.1 Study design and data source

A disproportionality analysis was conducted to quantify the correlation between roflumilast and psychiatric AEs by calculating the proportion of target AEs associated with roflumilast compared with all other pharmaceutical AEs (23). A significant signal emerged when roflumilast showed an increased likelihood of inducing a specific AE compared with all other drugs collectively. Data were extracted from the following website: https://fis.fda.gov/extensions/FPD-QDE-FAERS/FPD-QDE-FAERS.html (accessed May 2025). To include the most recent case reports, data from the first quarter of 2014 to the first quarter of 2025 were extracted.

### 2.2 Data extraction

Following FDA guidelines, a deduplication process was implemented to ensure the integrity and uniqueness of the dataset (24). Cases linked to roflumilast were identified using the search criteria “ROFLUMILAST” and “DALIRESP”, with the role designation set to “primary suspected” (PS) (25). The Medical Dictionary for Regulatory Activities 26.0 (MedDRA 26.0) was employed to encode, categorize and localize the signals to analyze the specific System Organ Class (SOC) and preferred term (PT) involved in AE signals. The intricate process of data manipulation was carried out using Python 3.10 (Python Software Foundation, Holland) and R 4.3.1 (R Foundation for Statistical Computing, Vienna, Austria), both of which contributed to the careful processing of the data.

**Figure 1:**
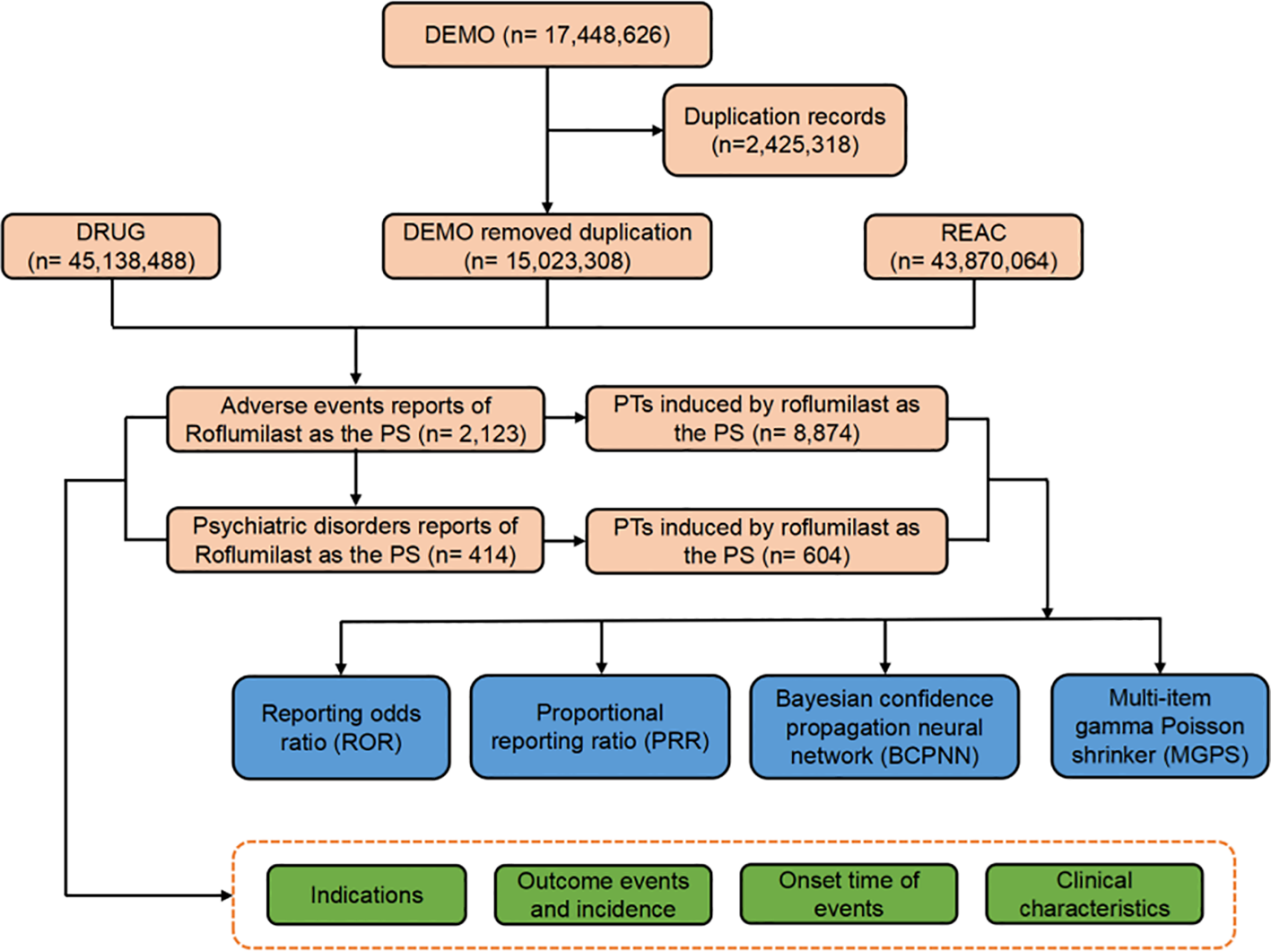
The flow diagram of selecting roflumilast-related AEs from the FAERS database.

A series of descriptive analysis were performed to provide a comprehensive overview of the clinical features in roflumilast-associated psychiatric AE’s reports. Where data permitted, detailed information was extracted and presented, covering patient characteristics (such as age and weight), severe outcomes manifestation, geographic distributions of reports, specific medical indications, and reporter identities. The analytical process culminated in the creation of a visually informative flowchart (**Figure 1**).

### 2.3 Data analysis

A 2 × 2 table was employed for disproportionality analysis (**Supplementary Table 1**), a method widely used in pharmacovigilance studies. Signals’ identification relies on the simultaneous utilization of four distinct algorithms: the reporting odds ratio (ROR), the proportional reporting ratio (PRR), the information component (IC), and the empirical Bayesian geometric mean (EBGM) (26). In our current investigation, an AE was classified as a signal only when all four algorithms met the predefined criteria. The precise equations and criteria governing the application of these four algorithms are presented in **Supplementary Table 2**, facilitating a comprehensive understanding of their respective applications.

### 2.4 Subgroup analysis

Subgroup analysis were performed to determine the differences between psychiatric AE reports and overall AE reports of roflumilast, and to evaluate the risk of psychiatric AEs occurring. The evaluation of psychiatric AE reports was undertaken through a systematic stratification by sex, age, weight, reported countries, indications, combination drugs, outcomes, and the nature of reporters.

### 2.5 Statistical analysis

For categorical variables within AEs, we provide frequencies alongside their corresponding percentages. Non-normally distributed data are depicted as median values with their interquartile range (IQR). Significance levels were set at a two-tailed threshold of *P* < 0.05. The Student’s t-test and Pearson chi-square test were used to assess statistical associations.

## 3 Results

### 3.1 Descriptive analysis

From the first quarter of 2014 to the first quarter of 2025, the FAERS database cataloged a total of 17, 448, 626 AE reports. Following the meticulous exclusion of duplicates, 2, 123 reports were identified as pertaining to roflumilast. Of these, 414 reports were distinctly associated with psychiatric AEs. The basic characteristics of patients with roflumilast-associated psychiatric AEs were summarized in **Table 1**.

**Table 1:** Clinical characteristics of reports with roflumilast from the FAERS database.

Characteristics	Roflumilast-induced AE reports (n = 414)		
Number of events	Available number, n	Case number, n	Case proportion, %
Gender, n (%)	378	–	91.30%
Female	–	225	59.52%
Male	–	153	40.48%
Age (years), n (%)	280	–	67.63%
< 18	–	0	0.00%
18 ≤ and ≤ 65	–	114	40.71%
> 65	–	166	59.29%
Median (IQR)	–	69 (62 - 75)	–
Weight (Kg), n (%)	202	–	48.79%
< 80	–	134	66.34%
80 ≤ and ≤ 100	–	54	26.73%
> 100	–	14	6.93%
Median (IQR)	–	70 (59 - 84)	–
Reported countries, n (%)	413	–	99.76%
US	–	359	86.92%
CA	–	40	9.69%
DE	–	7	1.69%
Other country	–	7	1.69%
Indications, n (%)	317	–	76.57%
COPD	–	242	76.34%
Asthma	–	56	17.67%
Dyspnoea	–	28	8.83%
Combination drugs, n (%)	292	–	70.53%
Salbutamol	–	161	55.14%
Tiotropium	–	136	46.58%
Budesonide/formoterol	–	101	34.59%
Outcomes, n (%)	414	–	100.00%
Non-serious Outcome	–	156	37.68%
Serious Outcome ^a^	–	258	62.32%
Death	–	19	7.36%
Life-threatening	–	24	9.30%
Hospitalization	–	109	42.25%
Disability	–	4	1.55%
Other serious outcomes	–	190	73.64%
Reporters, n (%)	338	–	81.64%
Health professional	–	170	50.30%
Consumer	–	168	49.70%
Time-to-onset (days)	38	–	9.18%
Median (IQR)		4.5 (0-14)	–
Reporting year, n (%)	414	–	100.00%
2014	–	58	14.01%
2015	–	36	8.70%
2016	–	46	11.11%
2017	–	56	13.53%
2018	–	39	9.42%
2019	–	50	12.08%
2020	–	21	5.07%
2021	–	15	3.62%
2022	–	40	9.66%
2023	–	22	5.31%
2024	–	21	5.07%
2025 ^b^	–	10	2.42%

^a^ Total serious outcomes may exceed the total number of reported cases because some cases list more than one serious outcomes.

^b^ The first quarter of 2025.

AE, adverse event; FAERS, FDA Adverse Event Reporting System; COPD, chronic obstructive pulmonary disease; IQR, interquartile range.

**Figure 2:**
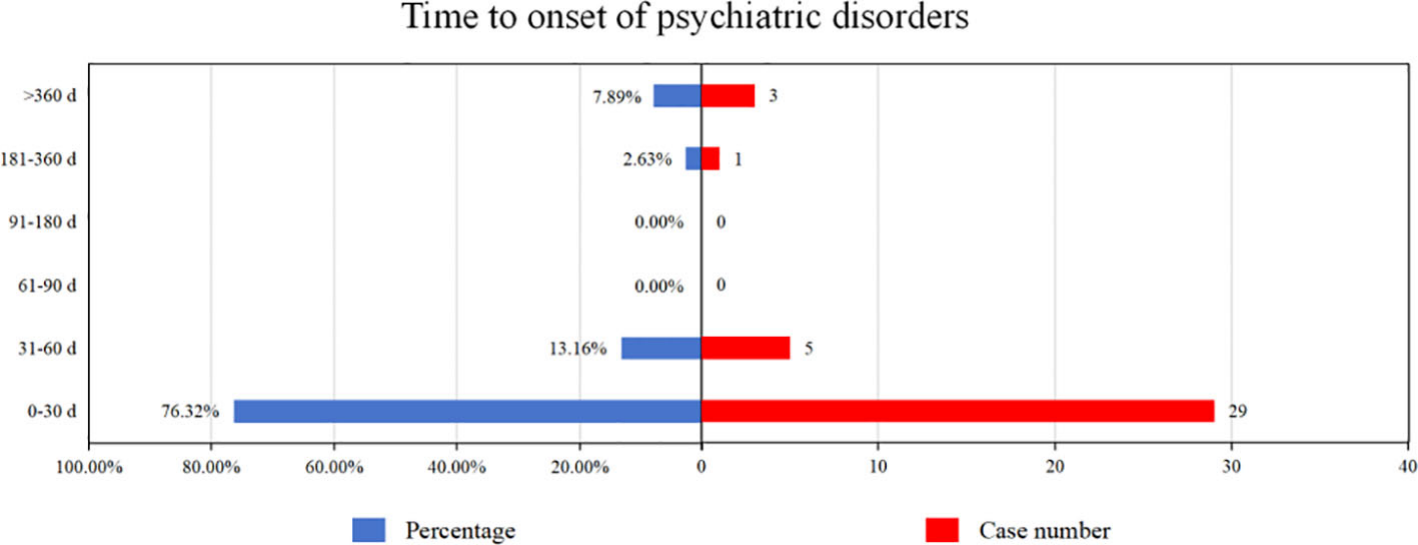
Time-to-onset of roflumilast-related psychiatric AEs.

Notably, a higher proportion of females was recorded among the psychiatric AE reports for which data were available, accounting for 59.52%, in contrast to 40.48% for males. Regarding age distribution, psychiatric AEs were more frequently documented in patients over 65 years old (59.29%), compared to patients aged 18 to 65 years old (40.71%), and patients under 18 years old (0.00%). The median age at which psychiatric AEs occurred was determined to be 69 years (interquartile range (IQR) 62–75 years). Most psychiatric AEs were reported in patients weighing less than 80 kg (66.34%), whereas patients weighing between 80 and 100 kg accounted for a smaller proportion of AE reports (26.73%). The median weight for psychiatric AEs was 70 kg (IQR: 59–84 kg). The majority of psychiatric AE reports originated from the USA (86.92%), trailed by Germany (9.69%) and Canada (1.69%). In terms of reported indications, COPD constituted more than half of the cases (76.34%), followed by other indications such as asthma (17.67%) and dyspnea (8.83%). The prevailing combination drugs in psychiatric AEs were salbutamol, tiotropium, and budesonide/formoterol. Notably, a significant proportion of psychiatric AE reports indicated serious outcomes (62.32%), with hospitalization (42.25%) being the predominant reported outcome, closely followed by cases resulting in life-threatening (9.30%) and death (7.36%). Healthcare professionals and consumers were responsible for the submitting the similar proportion of reported cases (50.30% and 49.70%, respectively). Furthermore, approximately 9.18% of patients reported the onset time of psychiatric AEs, revealing a median time-to-onset of 4.5 days (IQR 0–14 days). The AEs primarily occurred within 0–30 days (**Figure 2**), accounting for 76.32%.

### 3.2 Disproportionality analysis

Throughout the study, 31 distinct PTs associated with roflumilast-induced psychiatric AEs were discerned in the FAERS database, with each PT manifesting in at least three cases (**Table 2**). Among these, the most frequently reported psychiatric AEs were insomnia (n = 128), anxiety (n = 79), suicidal ideation (n = 49), depression (n = 40), and sleep disorder due to a general medical condition (n = 31). Five noteworthy signals relating to the PTs were emerged, encompassing sleep disorder due to a general medical condition (ROR 13.47), eating disorder (ROR 5.16), suicidal ideation (ROR 4.32), insomnia (ROR 3.55), and nervousness (ROR 3.30). The corresponding values for PRR, IC, and EBGM are depicted in **Table 2**.

**Table 2:** Signal strength of psychiatric AEs of roflumilast in FAERS database (n ≥ 3).

Preferred terms (PTs)	Number	ROR (95% CI)	PRR (χ^2^)	IC (IC025)	EBGM (EBGM05)
*Insomnia ^a^*	**128**	**3.55 (2.98-4.22)**	**3.51 (230.48)**	**1.81 (1.52)**	**3.49 (3.01)**
Anxiety	79	2.03 (1.63-2.54)	2.02 (41.03)	1.02 (0.81)	2.01 (1.66)
*Suicidal ideation ^a^*	**49**	**4.32 (3.27-5.73)**	**4.31 (124.41)**	**2.11 (1.59)**	**4.26 (3.35)**
Depression	40	1.38 (1.01-1.88)	1.38 (4.13)	0.46 (0.34)	1.36 (1.04)
*Sleep disorder due to a general medical condition ^a^*	**31**	**13.47 (9.46-19.17)**	**13.42 (355.55)**	**3.74 (2.63)**	**13.17 (9.69)**
*Nervousness ^a^*	**22**	**3.30 (2.17-5.01)**	**3.29 (35.07)**	**1.72 (1.13)**	**3.21 (2.22)**
Confusional state	19	0.88 (0.56-1.38)	0.88 (0.31)	-0.19 (-0.29)	0.86 (0.57)
*Eating disorder ^a^*	**16**	**5.16 (3.16-8.43)**	**5.15 (53.51)**	**2.36 (1.45)**	**4.99 (3.23)**
Sleep disorder	16	1.56 (0.95-2.54)	1.56 (3.19)	0.64 (0.39)	1.51 (0.98)
Depressed mood	13	1.70 (0.99-2.93)	1.70 (3.74)	0.76 (0.44)	1.63 (1.00)
Hallucination	11	1.12 (0.62-2.02)	1.12 (0.14)	0.16 (0.09)	1.07 (0.63)
Panic attack	11	2.34 (1.30-4.23)	2.34 (8.44)	1.23 (0.68)	2.23 (1.31)
Drug abuse	11	0.81 (0.45-1.46)	0.81 (0.51)	-0.31 (-0.56)	0.77 (0.45)
Mental disorder	9	1.55 (0.81-2.99)	1.55 (1.78)	0.64 (0.33)	1.47 (0.81)
Suicide attempt	9	1.21 (0.63-2.32)	1.21 (0.32)	0.27 (0.14)	1.14 (0.63)
Nightmare	8	1.79 (0.89-3.58)	1.79 (2.78)	0.84 (0.42)	1.68 (0.89)
Restlessness	7	1.47 (0.70-3.08)	1.47 (1.04)	0.55 (0.26)	1.36 (0.69)
Agitation	7	0.78 (0.37-1.63)	0.78 (0.46)	-0.37 (-0.77)	0.72 (0.37)
Stress	6	0.58 (0.26-1.30)	0.58 (1.79)	-0.78 (-1.73)	0.54 (0.25)
Abnormal dreams	5	1.72 (0.71-4.12)	1.72 (1.49)	0.78 (0.32)	1.55 (0.68)
Completed suicide	5	0.48 (0.20-1.16)	0.48 (2.80)	-1.05 (-2.53)	0.43 (0.19)
Mood altered	5	1.38 (0.57-3.31)	1.38 (0.52)	0.46 (0.19)	1.24 (0.54)
Mood swings	5	1.23 (0.51-2.96)	1.23 (0.22)	0.30 (0.11)	1.23 (0.48)
Anger	5	1.17 (0.48-2.80)	1.17 (0.12)	0.22 (0.09)	1.05 (0.46)
Psychotic disorder	4	1.13 (0.42-3.02)	1.13 (0.06)	0.18 (0.07)	1.99 (0.39)
Disorientation	4	0.84 (0.32-2.24)	0.84 (0.12)	-0.25 (-0.67)	0.74 (0.29)
Aggression	4	0.70 (0.26-1.85)	0.70 (0.53)	-0.52 (-1.39)	0.91 (0.24)
Schizophrenia	4	2.19 (0.82-5.83)	2.19 (2.58)	1.13 (0.42)	1.92 (0.75)
Thinking abnormal	4	1.72 (0.65-4.60)	1.72 (1.22)	0.79 (0.29)	1.51 (0.59)
Poor quality sleep	4	1.27 (0.48-3.38)	1.27 (0.22)	0.34 (0.13)	1.11 (0.43)
Fear	4	1.38 (0.52-3.68)	1.38 (0.42)	0.47 (0.17)	1.21 (0.47)
Personality change	3	2.67 (0.86-8.27)	2.67 (3.12)	1.41 (0.46)	2.23 (0.73)
Sleep disorder due to general medical condition, insomnia type	3	2.97 (0.96-9.22)	2.97 (3.92)	1.57 (0.51)	2.49 (0.81)
Irritability	3	0.38 (0.12-1.18)	0.38 (3.00)	-1.39 (-4.31)	0.21(0.10)
Middle insomnia	3	1.24 (0.40-3.86)	1.24 (0.14)	0.32 (0.10)	1.04 (0.34)
Drug dependence	3	0.10 (0.03-0.31)	0.10 (24.18)	-3.31 (-10.27)	0.08 (0.03)
Frustration tolerance decreased	3	1.66 (0.53-5.14)	1.66 (0.78)	0.73 (0.24)	1.39 (0.45)

The bold and italicized data indicated the four distinct algorithms’s results of positive signal.

^a^ Positive signal.

PTs, preferred terms, FAERS, FDA Adverse Event Reporting System; ROR, reporting odds ratio; CI, confidence interval; PRR, proportional reporting ratio; χ^2^, chi-squared; IC, information component; EBGM, empirical Bayesian geometric mean.

### 3.3 Subgroup analysis

Discernible distinctions in the clinical attributes of psychiatric AE reports in comparison to non-psychiatric AE reports of roflumilast are presented in **Table 3**. Psychiatric AE reports were evaluated through systematic stratification by sex, age, weight, reported countries, indications, combination drugs, outcomes, and the nature of reporters. Several notable observations were revealed: (1) A relatively high risk of psychiatric AEs in female patients (χ^2^ = 10.12, *P* = 0.001). (2) A relatively high risk of psychiatric AEs in persons aged 18-65 (χ^2^ = 25.55, *P* < 0.001). (3) More psychiatric AEs were reported in Canada than non-psychiatric AEs (χ^2^ = 9.05, *P* = 0.003). (4) Patients diagnosed with asthma were found to be at increased risk of experiencing psychiatric AEs (χ^2^ = 5.38, *P* = 0.020). (5) The inclusion of salbutamol (χ^2^ = 38.72, *P* < 0.001) and tiotropium (χ^2^ = 33.25, *P* < 0.001) in combination regimens appeared to be associated with a comparatively increased risk of psychiatric AEs. (6) A reduced propensity for serious outcomes was evident within the domain of psychiatric AEs (χ^2^ = 26.14, *P* < 0.001), particularly with regard to death (χ^2^ = 139.59, *P* < 0.001). While the risk of life-threatening (χ^2^ = 29.44, *P* < 0.001) and hospitalization (χ^2^ = 8.72, *P* = 0.003) increased relatively compared to non-psychiatric AEs. The time-to-onset of psychiatric AEs occurs earlier than non-psychiatric AEs (*P* < 0.001). (7) In addition, no significant disparities were observed concerning weight (*P* > 0.1) or qualifications of the reporters (*P* = 0.366).

**Table 3:** Differences in clinical characteristics of psychiatric AEs and non-psychiatric AEs reports.

Subgroup	Psychiatric AEs (n=414)	Non-psychiatric AEs (n=1,709)	Statistics ^b^	*p* value ^c^
Gender, n (%)				
Female	225 (59.52)	729 (50.35)	10.12	**0.001**
Male	153 (40.48)	719 (49.65)		
Age (years), n (%)				
18 ≤ and ≤ 65	114 (40.71)	235 (24.11)	25.55	**< 0.001**
> 65	166 (59.29)	700 (74.79)		
Weight (Kg), n (%)				
< 80	134 (66.34)	344 (66.93)	0.02	0.880
80 ≤ and ≤ 100	54 (26.73)	118 (22.96)	1.13	0.287
> 100	14 (6.93)	52 (10.12)	1.76	0.185
Reported countries, n (%)				
US (United States)	359 (86.92)	1,459 (87.37)	0.06	0.810
CA (Canada)	40 (9.69)	94 (5.63)	9.05	**0.003**
DE (Germany)	7 (1.69)	26 (1.56)	0.04	0.841
Indications, n (%)				
COPD	242 (76.34)	686 (78.85)	0.86	0.354
Asthma	56 (17.67)	108 (12.41)	5.38	**0.020**
Dyspnoea	28 (8.83)	51 (5.86)	3.30	0.069
Combination drugs, n (%)				
Salbutamol	161 (55.14)	308 (34.61)	38.72	**< 0.001**
Tiotropium	136 (46.58)	252 (28.31)	33.25	**< 0.001**
Budesonide/formoterol	101 (34.59)	269 (30.59)	1.95	0.163
Outcomes, n (%)				
Non-serious Outcome	156 (37.68)	430 (15.16)	26.14	**< 0.001**
Serious Outcomes ^a^	258 (62.32)	1,279 (74.84)		
Death	19 (7.36)	600 (46.91)	139.59	**< 0.001**
Life-threatening	24 (9.30)	31 (2.42)	29.44	**< 0.001**
Hospitalization	109 (42.25)	418 (32.68)	8.72	**0.003**
Disability	4 (1.55)	11 (0.86)	1.06	0.304
Time-to-onset (days)				
Median (IQR)	4.5 (0-14)	34 (3-118)	–	**< 0.001** ^d^
Reporters, n (%)				
Health professional	170 (50.30)	683 (47.56)	0.82	0.366
Consumer	168 (49.70)	753 (52.44)		

The bold data indicated statistical significance

^a^Total serious outcomes may exceed the total number of reported cases because some cases list more than one serious outcomes.

^b^The Pearson chi-square test, chi-squared value (χ**^2^**).

^c^P value, two-tailed threshold of p< 0.05.

^d^Student’s T test.

AE, adverse event; FAERS, the FDA Adverse Event Reporting System; IQR, interquartile rang.

## 4 Discussion

Rare yet serious psychiatric AEs have been reported in the COPD patients, with suicidality also emerging in clinical trials (13). The previous study reported that five patients receiving roflumilast experienced suicide-related AEs (18). Strikingly, the association between roflumilast and psychiatric disorders has scarcely been investigated over the past decade. In the present study, a pharmacovigilance approach was adopted to systematically explore the complex relationship between roflumilast use and psychiatric AEs, thereby determining its post-marketing safety profile. Our study identified 31 PTs associated with roflumilast-induced psychiatric AEs, five of which exhibited significant positive signals.

Patients with COPD are themselves at increased risk of developing psychiatric disorders, including depression, anxiety, and sleep disturbances (27, 28). Previous studies have demonstrated that chronic dyspnea, systemic inflammation, reduced physical activity, impaired quality of life, hypoxemia, and frequent exacerbations may contribute to psychiatric morbidity in COPD patients. Therefore, the psychiatric AEs observed in the present study may partially reflect the underlying psychiatric vulnerability associated with COPD itself, rather than being solely attributable to roflumilast exposure. Because the FAERS database lacks detailed clinical information regarding baseline psychiatric status and disease severity, residual confounding cannot be completely excluded. Consequently, the associations identified in this study should be interpreted cautiously.

In the present study, insomnia (n = 128, ROR = 3.55) was the most frequently reported psychiatric AEs associated with roflumilast, which is consistent with previous studies (17). Although its underlying mechanism remains unclear, our study and earlier studies consistently indicate that roflumilast is associated with the occurrence of insomnia. Insomnia is associated with an increased risk of developing various mental health disorders (29–31), including anxiety and depression (32, 33), which also appeared in our study. Although they were not positive signals, anxiety and depression were still common psychiatric AEs associated with roflumilast, with 79 and 40 case reports, respectively. Decreased appetite has been reported in previous studies (15–17), but was not observed in the present study. However, eating disorder (n = 16, ROR = 5.16) was identified as a positive signal. The mechanisms underlying eating disorder and weight loss associated with roflumilast may involve phosphodiesterase-4 (PDE4) inhibition-mediated increases in intracellular cyclic adenosine monophosphate (cAMP) levels (34, 35). Elevated cAMP signaling may alter the activation and phosphorylation state of AMP-activated protein kinase, thereby affecting lipolysis, energy expenditure, and appetite regulation. In addition, roflumilast may increase glucagon-like peptide-1 levels, which are associated with reduced appetite and enhanced satiety (36). Because appetite suppression and weight loss may adversely affect patients with low body mass index and advanced COPD, clinicians should closely monitor nutritional status during treatment (20). Weight loss may be an issue for patients with lower body mass; as prognosis is negatively affected in patients with a low body mass in the later stages of COPD (20). Nervousness (n = 22, ROR = 3.3) was not mentioned in previous studies or the drug label. It is one of the advantages of a post-marketing pharmacovigilance study to identify new and rare AEs, and this study has identified nervousness as such an AE. Therefore, health professionals should be alert to the symptoms of nervousness in patients using roflumilast so that treatment regimens can be adjusted in time to avoid serious consequences. Roflumilast is associated with several uncommon psychiatric AEs in relation to existing COPD therapies. Future studies of longer duration are necessary to determine the risk associated with the psychiatric AEs. Until more is known, patients should be monitored closely for psychiatric symptoms (17).

Notably, a total of 49 cases of suicidal ideation (ROR = 4.32) were reported among all roflumilast-related psychiatric AEs, which is a concerning number compared to case reports in clinical studies. Additionally, nine cases of suicide attempt and five cases of completed suicide were reported. Although these were not considered positive signals, they were equally merited attention from health professionals. A prior meta-analysis demonstrated that, although rare, suicide was more common in the roflumilast group than in the placebo group (19, 37). The FDA conducted a comprehensive analysis of possible suicide-related AEs in COPD patients treated with roflumilast using the Columbia Classification Algorithm of Suicide Assessment (C-CASA). Unexpectedly, the results revealed no new potential suicide-related AE (19), and the difference in suicidal events between roflumilast and placebo was not statistically significant. Nonetheless, for patients with a history of depression or suicidal thoughts/behavior, the risk-benefit profile of roflumilast therapy must be carefully weighed, and they must be monitored vigilantly for any emerging or worsening psychiatric symptoms (13).

Medications prescribed for chronic diseases can lead to short-term psychiatric symptoms, yet their long-term impact on psychiatric conditions remain unclear (38). Until now, the mechanisms underlying psychiatric AEs associated with roflumilast have not been fully elucidated. As a selective PDE4 inhibitor, roflumilast suppresses the degradation of intracellular cAMP, thereby increasing intracellular cAMP concentrations. PDE4 is widely expressed in inflammatory cells as well as in multiple regions of the central nervous system, including the hippocampus, amygdala, and prefrontal cortex, which are critically involved in emotional regulation and sleep physiology (34). Alterations in cAMP signaling pathways may influence neurotransmitter release, synaptic plasticity, neuroimmune responses, and HPA axis activity, potentially contributing to psychiatric symptoms such as insomnia, anxiety, depression, and suicidal ideation (35, 39). Previous studies have shown that abnormal cAMP signaling may affect serotonergic, dopaminergic, and glutamatergic neurotransmission, all of which play important roles in mood regulation and emotional processing (35). In addition, PDE4 inhibition may influence sleep-wake regulation through modulation of neuronal excitability and circadian signaling pathways, potentially contributing to the high frequency of insomnia observed in this study. Furthermore, oral medications may alter gut microbiota composition, thereby affecting brain function through the microbiota-gut-brain axis. Gut microbiota can regulate central nervous system activity through immune, endocrine, and neural pathways, including the production of tryptophan metabolites and neurotransmitter precursors (40–45). Numerous studies have demonstrated that gut dysbiosis is associated with depression, anxiety, and stress-related neuropsychiatric disorders (40, 44, 45). Because diarrhea and gastrointestinal AEs are common with roflumilast treatment, alterations in intestinal microbiota may partially contribute to psychiatric AEs. However, the precise biological mechanisms remain unclear and warrant further mechanistic and prospective investigations.

Subgroup analysis revealed several differences between psychiatric and non-psychiatric AEs (**Table 3**). The relatively higher risk of psychiatric AEs in female patients receiving roflumilast (*P* = 0.001) is consistent with a previous study suggesting that females are more susceptible to psychiatric disorders (46). The previous study revealed that neither age nor sex affected the relationship between insomnia (47). However, psychiatric morbidity may have a greater impact on females than on males, which could elevate the risk of suicide among females (48, 49). Therefore, health professionals should pay closer attention to suicidal events in female patients experiencing psychiatric AEs, particularly those with a history of depression and/or suicidal thoughts or behavior. In an analysis of roflumilast use in patients aged 65 years, AEs were found to occur more frequently (50). However, our analysis revealed that the risk of psychiatric AEs was higher in patients younger than 65 years (*P* < 0.001). Patients with asthma were more likely to have an antecedent psychiatric disorder prior to their first episode, particularly depressive disorders (51). The same results were observed in the present study, where asthmatics were found to be at a higher risk of experiencing psychiatric AEs (*P* = 0.020). The risk of psychiatric AEs was higher when roflumilast was combined with salbutamol or tiotropium (*P* < 0.001), possibly because salbutamol and tiotropium also induce psychiatric AEs (52, 53). This study found that psychiatric AEs were associated with a lower risk of serious outcomes than non-psychiatric AEs. However, there was an increased risk of hospitalization and of life-threatening events. Deaths caused by non-psychiatric AEs may be attributable to disease progression, while it may be due to suicidal events in psychiatric AEs. Although psychiatric AEs appeared to occur earlier than non-psychiatric AEs, these findings should be interpreted cautiously because time-to-onset information was available for only a limited number of reports in the FAERS database, which may reduce the statistical robustness and generalizability of the analysis.

Although the FAERS database provides valuable real-world insights into rare and serious AEs, several inherent limitations should be acknowledged. First, the voluntary nature of FAERS reporting may introduce reporting bias, underreporting, selective reporting, and incomplete clinical information. In particular, data regarding age, body weight, comorbidities, baseline psychiatric history, COPD severity, and time-to-onset were unavailable for many reports, thereby limiting detailed subgroup analysis and causal interpretation. Second, COPD itself is associated with an increased risk of psychiatric disorders, including depression, anxiety, and sleep disturbances, which may act as important confounding factors in the present study (27, 28). Because FAERS lacks detailed longitudinal clinical data and untreated control populations, it is difficult to completely distinguish psychiatric symptoms related to roflumilast from those associated with the underlying disease. Third, spontaneous reports submitted by consumers may contain inaccuracies or inconsistencies because of limited medical expertise. Additionally, concomitant medications and multiple comorbid conditions may further complicate interpretation of the observed associations. Finally, although disproportionality analysis are useful for signal detection, they cannot establish causality. Therefore, our findings should be regarded as hypothesis-generating rather than confirmatory. Future large-scale prospective studies and mechanistic investigations are warranted to validate these findings and further elucidate the biological mechanisms underlying roflumilast-associated psychiatric AEs.

## 5 Conclusion

Utilizing the FAERS database, we conducted an analysis of post-marketing psychiatric AEs associated with roflumilast. This investigation unveiled potential psychiatric AEs, including insomnia, suicidal ideation, sleep disorder, nervousness, and eating disorder. Notably, psychiatric AEs may be related to a heightened risk of life-threatening and hospitalization. Our study furnishes healthcare professionals with invaluable insights, informing the implementation of strategies to mitigate the risk of psychiatric AEs associated with roflumilast administration.

## Data Availability

Publicly available datasets were analyzed in this study. This data can be found here: https://fis.fda.gov/extensions/FPD-QDE-FAERS/FPD-QDE-FAERS.html.

## References

[1] Wu X Jia B Luo X Wang J Li M . Glucocorticoid alleviates mechanical stress-induced airway inflammation and remodeling in COPD via transient receptor potential canonical 1 channel. Int J Chron Obstruct Pulmon Dis. (2023) 18:1837–1851. doi: 10.2147/copd.S419828 PMC10466112 37654522

[2] Subedi S Guntipally M Suwal N Thapa R Bashyal S Panth N . Cellular senescence in chronic obstructive pulmonary disease: molecular mechanisms and therapeutic interventions. Ageing Res Rev. (2025) 110:102813. doi: 10.1016/j.arr.2025.102813 40571131

[3] Parums DV . Editorial: Global Initiative for Chronic Obstructive Lung Disease (GOLD) 2023 guidelines for COPD, including COVID-19, climate change, and air pollution. Med Sci Monit. (2023) 29:e942672. doi: 10.12659/msm.942672 37777859 PMC10552569

[4] Wang Y Xu J Meng Y Adcock IM Yao X . Role of inflammatory cells in airway remodeling in COPD. Int J Chron Obstruct Pulmon Dis. (2018) 13:3341–3348. doi: 10.2147/copd.S176122 PMC6190811 30349237

[5] Bundschuh DS Eltze M Barsig J Wollin L Hatzelmann A Beume R . In vivo efficacy in airway disease models of roflumilast, a novel orally active PDE4 inhibitor. J Pharmacol Exp Ther. (2001) 297:280–290. doi: 10.1016/s0022-3565(24)29538-0 11259555

[6] Hatzelmann A Schudt C . Anti-inflammatory and immunomodulatory potential of the novel PDE4 inhibitor roflumilast in vitro. J Pharmacol Exp Ther. (2001) 297:267–279. doi: 10.1016/S0022-3565(24)29537-9 11259554

[7] Spina D . PDE4 inhibitors: current status. Br J Pharmacol. (2008) 155:308–15. doi: 10.1038/bjp.2008.307 18660825 PMC2567892

[8] Grootendorst DC Gauw SA Verhoosel RM Sterk PJ Hospers JJ Bredenbröker DM . Reduction in sputum neutrophil and eosinophil numbers by the PDE4 inhibitor roflumilast in patients with COPD. Thorax. (2007) 62(12):1081–1087. doi: 10.1136/thx.2006.075937 PMC2094292 17573446

[9] Fabbri LM Calverley PM Izquierdo-Alonso JL Bundschuh DS Brose M Martinez FJ . Roflumilast in moderate-to-severe chronic obstructive pulmonary disease treated with longacting bronchodilators: two randomised clinical trials. Lancet. (2009) 374(9691):695–703. doi: 10.1016/s0140-6736(09)61252-6 19716961

[10] Calverley PM Rabe KF Goehring UM Kristiansen S Fabbri LM Martinez FJ . Roflumilast in symptomatic chronic obstructive pulmonary disease: two randomised clinical trials. Lancet. (2009) 374(9691):685–694. doi: 10.1016/s0140-6736(09)61255-1 19716960

[11] Martinez FJ Calverley PM Goehring UM Brose M Fabbri LM Rabe KF . Effect of roflumilast on exacerbations in patients with severe chronic obstructive pulmonary disease uncontrolled by combination therapy (REACT): a multicentre randomised controlled trial. Lancet. (2015) 385(9971):857–866. doi: 10.1016/s0140-6736(14)62410-7 25684586

[12] Rabe KF Bateman ED O'Donnell D Witte S Bredenbröker D Bethke TD . Roflumilast--an oral anti-inflammatory treatment for chronic obstructive pulmonary disease: a randomised controlled trial. Lancet. (2005) 366(9485):563–571. doi: 10.1016/s0140-6736(05)67100-0 16099292

[13] FDA-label-roflumilast. Available online at: https://nctr-crs.fda.gov/fdalabel/services/spl/set-ids/ca22f3e0-6f33-4f1c-97e5-0c962891067a/spl-doc?hl=roflumilast. (Accessed May 24, 2025).

[14] Garnock-Jones KP . Roflumilast: a review in COPD. Drugs. (2015) 75:1645–56. doi: 10.1007/s40265-015-0463-1 26338438

[15] Martinez FJ Rabe KF Calverley PMA Fabbri LM Sethi S Pizzichini E . Determinants of response to roflumilast in severe chronic obstructive pulmonary disease. Pooled analysis of two randomized trials. Am J Respir Crit Care Med. (2018) 198(10):1268–1278. doi: 10.1164/rccm.201712-2493OC 29763572

[16] Calverley PM Sanchez-Toril F McIvor A Teichmann P Bredenbroeker D Fabbri LM . Effect of 1-year treatment with roflumilast in severe chronic obstructive pulmonary disease. Am J Respir Crit Care Med. (2007) 176(2):154–161. doi: 10.1164/rccm.200610-1563OC 17463412

[17] Pinner NA Hamilton LA Hughes A . Roflumilast: a phosphodiesterase-4 inhibitor for the treatment of severe chronic obstructive pulmonary disease. Clin Ther. (2012) 34:56–66. doi: 10.1016/j.clinthera.2011.12.008 22284994

[18] Gross N Calverly PM Fabbri LM Rabe KF Mosberg H . Characterization of safety with roflumilast, an oral phosphodiesterase-4 inhibitor for the treatment of COPD. Chest. (2010) 138(4):466A. doi: 10.1378/chest.10494

[19] Oba Y Lone NA . Efficacy and safety of roflumilast in patients with chronic obstructive pulmonary disease: a systematic review and meta-analysis. Ther Adv Respir Dis. (2013) 7:13–24. doi: 10.1177/1753465812466167 23197074

[20] Janjua S Fortescue R Poole P . Phosphodiesterase-4 inhibitors for chronic obstructive pulmonary disease. Cochrane Database Syst Rev. (2020) 5:Cd002309. doi: 10.1002/14651858.CD002309.pub6 32356609 PMC7193764

[21] Sakaeda T Kadoyama K Okuno Y . Adverse event profiles of platinum agents: data mining of the public version of the FDA adverse event reporting system, AERS, and reproducibility of clinical observations. Int J Med Sci. (2011) 8:487–91. doi: 10.7150/ijms.8.487 21897761 PMC3167097

[22] Michel C Scosyrev E Petrin M Schmouder R . Can disproportionality analysis of post-marketing case reports be used for comparison of drug safety profiles? Clin Drug Investig. (2017) 37(5):415–422. doi: 10.1007/s40261-017-0503-6 28224371

[23] Ke C Chen M Lin L Huang Y . Postmarketing adverse events of tamoxifen in male and female patients with breast cancer. Int J Cancer. (2025) 156(4):734–743. doi: 10.1002/ijc.35193 39305475

[24] Zhang Q Ding Y Shu Y Chen J . A real-world disproportionality analysis of Rucaparib: post-marketing pharmacovigilance data. BMC Cancer. (2023) 23(1):745. doi: 10.1186/s12885-023-11201-w 37568126 PMC10416473

[25] Shu Y Ding Y Liu L Zhang Q . Cardiac adverse events associated with quetiapine: disproportionality analysis of FDA adverse event reporting system. CNS Neurosci Ther. (2023) 29(10):2705–2716. doi: 10.1111/cns.14215 PMC10401141 37032639

[26] Forbes SA Bindal N Bamford S Cole C Kok CY Beare D . COSMIC: mining complete cancer genomes in the Catalogue of Somatic Mutations in Cancer. Nucleic Acids Res. (2011) 39(Database issue):D945–D950. doi: 10.1093/nar/gkq929 PMC3013785 20952405

[27] Yohannes AM Alexopoulos GS . Depression and anxiety in patients with COPD. Eur Respir Rev. (2014) 23:345–9. doi: 10.1183/09059180.00007813 25176970 PMC4523084

[28] Ouellette DR Lavoie K . Recognition, diagnosis, and treatment of cognitive and psychiatric disorders in patients with COPD. Int J Chron Obstruct Pulmon Dis. (2017) 12:639–650. doi: 10.2147/copd.S123994 PMC5317263 28243081

[29] Hertenstein E Benz F Schneider CL Baglioni C . Insomnia-a risk factor for mental disorders. J Sleep Res. (2023) 32(6):e13930. doi: 10.1111/jsr.13930 37211915

[30] Freeman D Sheaves B Waite F Harvey AG Harrison PJ . Sleep disturbance and psychiatric disorders. Lancet Psychiatry. (2020) 7(7):628–637. doi: 10.1016/s2215-0366(20)30136-x 32563308

[31] Hertenstein E Feige B Gmeiner T Kienzler C Spiegelhalder K Johann A . Insomnia as a predictor of mental disorders: a systematic review and meta-analysis. Sleep Med Rev. (2019) 43:96–105. doi: 10.1016/j.smrv.2018.10.006 30537570

[32] Ahmadi R Rahimi-Jafari S Olfati M Javaheripour N Emamian F Ghadami MR . Insomnia and post-traumatic stress disorder: a meta-analysis on interrelated association (n = 57,618) and prevalence (n = 573,665). Neurosci Biobehav Rev. (2022) 141:104850. doi: 10.1016/j.neubiorev.2022.104850 36058403

[33] Marino C Andrade B Montplaisir J Petit D Touchette E Paradis H . Testing bidirectional, longitudinal associations between disturbed sleep and depressive symptoms in children and adolescents using cross-lagged models. JAMA Netw Open. (2022) 5(9):e2227119. doi: 10.1001/jamanetworkopen.2022.27119 35994289 PMC9396361

[34] Houslay MD Schafer P Zhang KY . Keynote review: phosphodiesterase-4 as a therapeutic target. Drug Discov Today. (2005) 10:1503–19. doi: 10.1016/s1359-6446(05)03622-6 16257373

[35] Zhang HT . Cyclic AMP-specific phosphodiesterase-4 as a target for the development of antidepressant drugs. Curr Pharm Des. (2009) 15:1688–98. doi: 10.2174/138161209788168092 19442182

[36] Rabe KF . Update on roflumilast, a phosphodiesterase 4 inhibitor for the treatment of chronic obstructive pulmonary disease. Br J Pharmacol. (2011) 163:53–67. doi: 10.1111/j.1476-5381.2011.01218.x 21232047 PMC3085868

[37] Rogliani P Calzetta L Cazzola M Matera MG . Drug safety evaluation of roflumilast for the treatment of COPD: a meta-analysis. Expert Opin Drug Saf. (2016) 15(8):1133–1146. doi: 10.1080/14740338.2016.1199683 27279341

[38] Chen Z Wang X Teng Z Huang J Mo J Qu C . A comprehensive assessment of the association between common drugs and psychiatric disorders using Mendelian randomization and real-world pharmacovigilance database. EBioMedicine. (2024) 107:105314. doi: 10.1016/j.ebiom.2024.105314 39191171 PMC11400609

[39] O'Donnell JM Zhang HT . Antidepressant effects of inhibitors of cAMP phosphodiesterase (PDE4). Trends Pharmacol Sci. (2004) 25:158–63. doi: 10.1016/j.tips.2004.01.003 15019272

[40] Socała K Doboszewska U Szopa A Serefko A Włodarczyk M Zielińska A . The role of microbiota-gut-brain axis in neuropsychiatric and neurological disorders. Pharmacol Res. (2021) 172:105840. doi: 10.1016/j.phrs.2021.105840 34450312

[41] Mayer EA Nance K Chen S . The gut-brain axis. Annu Rev Med. (2022) 73:439–53. doi: 10.1146/annurev-med-042320-014032 34669431

[42] Dinan TG Cryan JF . The microbiome-gut-brain axis in health and disease. Gastroenterol Clin North Am. (2017) 46:77–89. doi: 10.1016/j.gtc.2016.09.007 28164854

[43] Stilling RM Dinan TG Cryan JF . Microbial genes, brain & behaviour - epigenetic regulation of the gut-brain axis. Genes Brain Behav. (2014) 13:69–86. doi: 10.1111/gbb.12109 24286462

[44] Foster JA McVey Neufeld KA . Gut-brain axis: how the microbiome influences anxiety and depression. Trends Neurosci. (2013) 36:305–12. doi: 10.1016/j.tins.2013.01.005 23384445

[45] Luna RA Foster JA . Gut brain axis: diet microbiota interactions and implications for modulation of anxiety and depression. Curr Opin Biotechnol. (2015) 32:35–41. doi: 10.1016/j.copbio.2014.10.007 25448230

[46] Lee R Lee SM Hong M Oh IH . Psychiatric health risks in North Korean refugee youths resettled in South Korea. JAMA Netw Open. (2025) 8(4):e2512941. doi: 10.1001/jamanetworkopen.2025.12941 40440014 PMC12123469

[47] Samea F Mortazavi N Reimann GM Ebneabbasi A Zarei M Khazaie H . Insomnia and emotion dysregulation: a meta-analytical perspective integrating regulatory strategies and dispositional difficulties. Sleep Med Rev. (2025) 82:102111. doi: 10.1016/j.smrv.2025.102111 40554329

[48] Nilsson SF Wimberley T Speyer H Hjorthøj C Fazel S Nordentoft M . The bidirectional association between psychiatric disorders and sheltered homelessness. Psychol Med. (2024) 54(4):742–752. doi: 10.1017/s0033291723002428 37679023

[49] Nilsson SF Laursen TM Erlangsen A Hawton K Nordentoft M Fazel S . Homelessness, psychiatric disorders, and risks of suicide and self-harm: a population-based cohort study. Lancet Public Health. (2025) 10(7):e559–e567. doi: 10.1016/s2468-2667(25)00100-8 40602855

[50] Hanania NA Dransfield MT Goehring UM Lakkis H Rowe P . Efficacy of roflumilast in elderly patients with chronic obstructive pulmonary disease. Am J Respir Crit Care Med. (2011) 183:A3087. doi: 10.1164/ajrccm-conference.2011.183.1_meetingabstracts.a3087

[51] Michel IM Gardea-Reséndez M Ercis M Ortiz-Orendain J Ali DN Pazdernik VK . Examining the moderating effect of sex on the association between asthma and age of onset in bipolar disorder and schizophrenia. J Acad Consult Liaison Psychiatry. (2025) 66(6):482–491. doi: 10.1016/j.jaclp.2025.06.002 PMC12530441 40532945

[52] Bokov P El Jurdi H Denjoy I Peiffer C Medjahdi N Holvoet L . Salbutamol worsens the autonomic nervous system dysfunction of children with sickle cell disease. Front Physiol. (2020) 11:31. doi: 10.3389/fphys.2020.00031 32174840 PMC7054439

[53] Robles-Hernández RE Montiel-Lopez F Velázquez-Uncal M Sansores RH Hernández-Zenteno RJ Pérez-Padilla R . Efficacy of indacaterol vs tiotropium in COPD patients due to biomass exposure in improving quality of life and reducing symptoms. Respir Med. (2025) 241:108074. doi: 10.1016/j.rmed.2025.108074 40169096

