# Supplementary material for: Psychiatric events induced by roflumilast: a real-world pharmacovigilance study of the FDA Adverse Event Reporting System database
Source: Front Psychiatry. 2026 Jun 11;17:1836593. doi: 10.3389/fpsyt.2026.1836593 (PMC13294471; doi:10.3389/fpsyt.2026.1836593)
Supplement: Supplementary file 1 [file SupplementaryFile1.docx]

**Table S1** Two-by-two contingency (2×2) table for disproportionality analyses.

|  | **Target adverse event** | **All other adverse events** | **total** |
| --- | --- | --- | --- |
| **Target drug** | a | b | a+b |
| **All other drugs** | c | d | c+d |
| **total** | a+c | b+d | a+b+c+d |

Equation: a, number of reports containing both the target drug and target adverse drug reaction; b, number of reports containing other adverse drug reaction of the target drug; c, number of reports containing the target adverse drug reaction of other drugs; d, number of reports containing other drugs and other adverse drug reactions.

**Table S2** algorithms used for signal detection.

| **Algorithms** | **Equation** | **Criteria** |
| --- | --- | --- |
| ROR | ROR = ad/c/b | ROR05 > 1, N ≥ 2 |
|  | 95%CI = e^ln(ROR)±1.96(1/a+1/b+1/c+1/d)^0.5^ |  |
| PRR | PRR = [a/(a+b)]/[c/(c + d)] | PRR≥2 |
|  | χ^2^ = [(ad-bc)^2^ (a+b + c + d)]/[(a+b)(c + d)(a+c)(b + d)] | χ^2^ ≥ 4, N ≥ 3 |
| BCPNN | IC = log2 [a (a+b + c + d)]/[(a+c)(a+b)] | IC025 > 0 |
|  | 95%CI = e^ln(IC)±1.96(1/a+1/b+1/c+1/d)^0.5^ |  |
| MGPS | EBGM = a (a+b + c + d)/(a+c)/(a+b) | EBGM05 > 2, N ≥ 0 |
|  | 95%CI = e^ln(EBGM)±1.96(1/a+1/b+1/c+1/d)^0.5^ |  |

Abbreviations: N, number of adverse event reports; CI, confidence interval; ROR, reporting odds ratio; ROR05, the lower limit of the 95 two-sided CI of the ROR; N, the number of co-occurrences; PRR, proportional reporting ratio; χ2, chi-squared; BCPNN, bayesian confidence propagation neural network; IC, information component; IC025, the lower limit of the 95 two-sided CI of the IC; MGPS, multi-item gamma Poisson shrinker; EBGM, empirical bayesian geometric mean; EBGM05, the lower 95 two-sided CI of EBGM.
